# The association between psychological factors and breastfeeding behaviour in women with a body mass index (BMI) ≥30 kg m^−2^: a systematic review

**DOI:** 10.1111/obr.12681

**Published:** 2018-03-24

**Authors:** S. Lyons, S. Currie, S. Peters, T. Lavender, D. M. Smith

**Affiliations:** ^1^ Manchester Centre for Health Psychology, Division of Psychology and Mental Health, School of Health Sciences, Faculty of Biology, Medicine and Health The University of Manchester Manchester UK; ^2^ Department of Psychology, Faculty of Natural Sciences University of Stirling Stirling UK; ^3^ Centre for Global Women's Health, Division of Nursing, Midwifery and Social Work, Faculty of Biology, Medicine and Health The University of Manchester Manchester UK; ^4^ School of Social and Health Sciences Leeds Trinity University Horsforth UK

**Keywords:** Breastfeeding, obesity, psychological factors, women with a BMI ≥30 kg m^−2^

## Abstract

Breastfeeding can play a key role in the reduction of obesity, but initiation and maintenance rates in women with a body mass index (BMI) of ≥30 kg m^−2^ are low. Psychological factors influence breastfeeding behaviours in the general population, but their role is not yet understood in women with a BMI ≥30 kg m^−2^. Therefore, this review aimed to systematically search and synthesize the literature, which has investigated the association between any psychological factor and breastfeeding behaviour in women with a BMI ≥30 kg m^−2^. The search identified 20 eligible papers, reporting 16 psychological factors. Five psychological factors were associated with breastfeeding behaviours: intentions to breastfeed, belief in breast milk's nutritional adequacy and sufficiency, belief about other's infant feeding preferences, body image and social knowledge. It is therefore recommended that current care should encourage women to plan to breastfeed, provide corrective information for particular beliefs and address their body image and social knowledge. Recommendations for future research include further exploration of several psychological factors (i.e. expecting that breastfeeding will enhance weight loss, depression, anxiety and stress) and evidence and theory‐based intervention development.

## Introduction

Breastfeeding is associated with copious health benefits for both mother and child 1. In particular, breastfeeding can play a key role in the reduction and prevention of obesity 2, 3, 4. Therefore, the World Health Organization (WHO) recommends that all mothers should exclusively breastfeed their infants until they reach 6 months of age and continue with complementary breastfeeding until they reach at least 2 years 5.

However, adherence in women with a body mass index (BMI) of ≥30 kg m^−2^ is consistently low; women with a BMI ≥30 kg m^−2^ are less likely to initiate breastfeeding and more likely to breastfeed for shorter durations than their normal weight counterparts (BMI 18–24.99 kg m^−2^) 6, 7. Children born to women with a BMI ≥30 kg m^−2^ are at an increased risk of becoming obese 8, 9 and developing associated diseases (e.g. diabetes, hypertension and dyslipidaemia) 10. As breastfed infants experience a considerable reduction in risk of obesity and disease 4, 11, it is vital that we investigate the factors that influence breastfeeding practices in women with a BMI ≥30 kg m^−2^, in order to increase these behaviours and, ultimately, reduce the prevalence of obesity and related diseases.

Psychological factors (i.e. factors that affect or arise in an individual's mind) 12 have been consistently shown to influence breastfeeding behaviours in the general population 13, 14. For example, correlational studies have associated several psychological factors (e.g. perceived paternal support, confidence, dispositional optimism, breastfeeding expectations, faith in breast milk and knowledge) with increased breastfeeding initiation (i.e. beginning breastfeeding shortly after birth), duration (i.e. maintaining breastfeeding over a period of time) or exclusivity (i.e. giving the infant only breast milk) 13, 14. Likewise, several intervention studies 15, 16, 17 have shown that increasing self‐efficacy, knowledge and support can increase breastfeeding initiation and duration. This suggests, therefore, that psychological factors may be useful for increasing breastfeeding behaviours (i.e. initiation and duration).

Furthermore, studies have reported a positive association between psychological factors and breastfeeding behaviours specifically in women with a BMI ≥30 kg m^−2^ 18, 19, 20. This suggests that developing interventions that utilize psychological factors may be a successful method to increase breastfeeding initiation and duration in this population. An emerging literature examines interventions, which aim to increase breastfeeding rates in women with a BMI ≥30 kg m^−2^ 21, 22, 23, but only one study has reported benefits of a short increase in duration 22. However, this sample was not typical (i.e. participants were highly educated and likely highly motivated) limiting the generalizability of these results 24. A Cochrane review to examine interventions to support breastfeeding behaviour in women with a BMI ≥30 kg m^−2^ is underway 25 but proposes to focus on education, social support or physical interventions, rather than psychological factors and approaches. Systematic investigation of psychological factors that influence women's breastfeeding behaviours will inform the design of behavioural models of breastfeeding and public health interventions, to improve breastfeeding rates in this population and, ultimately, the long‐term health of women with BMI ≥30 kg m^−2^ and their children. Therefore, this review aimed to systematically search and synthesize the literature, which has investigated the association between, or the direct effect of, any psychological factor on breastfeeding behaviour in women with a BMI ≥30 kg m^−2^. The research question was ‘which psychological factors are associated with breastfeeding behaviours in women with a BMI ≥30kg/m^2^?’

## Methods

This review is reported in the style of the Checklist of Items to Include When Reporting a Systematic Review or Meta‐Analysis 26. The protocol was published on PROSPERO on 9 November 2016 (http://www.crd.york.ac.uk/PROSPERO/display_record.asp?ID=CRD42016050997).

### Eligibility criteria

This review included studies that investigated the association between or the direct effect of any psychological factor(s) on breastfeeding initiation and duration in women with a BMI ≥30 kg m^−2^. The eligibility criteria were specified according to the PICO framework (Table 1), stated in the preferred reporting items for systematic review and meta‐analysis statement 26.

**Table 1:** Inclusion criteria

PICO reference	Inclusion criteria
Population	Pre‐pregnancy BMI ≥30 kg m^−2^
	Live birth
	Opportunity to initiate/maintain breastfeeding
Intervention	Not used
Comparison	Not used
Outcome	Psychological factors (measured quantitatively)
Study	Prospective
	Cross‐sectional
	Intervention

BMI, body mass index.

Because of funding restrictions, all included studies were written in English. No restrictions were placed on date. Psychological factors were defined as any factor that affects or arises in an individual's mind 12. The population was women with a BMI ≥30 kg m^−2^ (WHO classification of obesity) 27, who have had a live birth, and the opportunity to initiate (i.e. begin shortly after birth) and maintain breastfeeding (i.e. continue to any extent). Studies were included if they included any quantitative baseline measure of at least one psychological factor and then reported subsequent breastfeeding behaviours (e.g. initiation or duration of any breastfeeding), measured psychological factors and the rate of breastfeeding within the sample and reported a direct correlation between a psychological factor and breastfeeding behaviour. As any measure of a psychological factor was permitted, there was no principal summary measure. Intervention studies were only included if they reported separate and individually measured psychological factors. Studies that pooled analyses between BMI categories were only included if the average BMI of the sample was ≥30 kg m^−2^. Only studies that reported using pre‐pregnancy BMI to determine weight status were included. Qualitative papers that addressed the research question were reviewed separately.

### Search strategy

Following a scoping exercise to finalize suitable search terms, an electronic systematic search of the literature using multi‐field search builders was conducted in PsycINFO, PubMed and Cumulative Index to Nursing and Allied Health Literature databases in August 2017. Grey literature was searched on OpenGrey, MedNar and Trove, and hand searching of journals and authors was conducted for included studies. Search terms were generated by conducting a scoping exercise in each database and with the use of Medical Subject Headings (Table 2).

**Table 2:** Keywords for each search term

PICO reference	Term	Keywords
P	Breastfeeding	Breastfe*, breast fe*, lactat* and infant feeding
	BMI ≥30 kg m^−2^	Obes*, body mass index, bmi, body mass index 30, bmi 30 and overweight
O	Psychological factors	psychosocial factors, psychological, social, social norms, social support, psychosocial support, self‐efficacy, expectations, education, health education, well being, wellbeing, psychological well‐being, body image, confidence, self‐confidence, knowledge, health knowledge, motivation, views, self‐esteem, self‐perception, attitudes, beliefs, postpartum depression, anxiety, stress, psychological stress, social acceptance and social influence

* Represents truncation.

BMI, body mass index.

### Study selection

**Figure 1:**
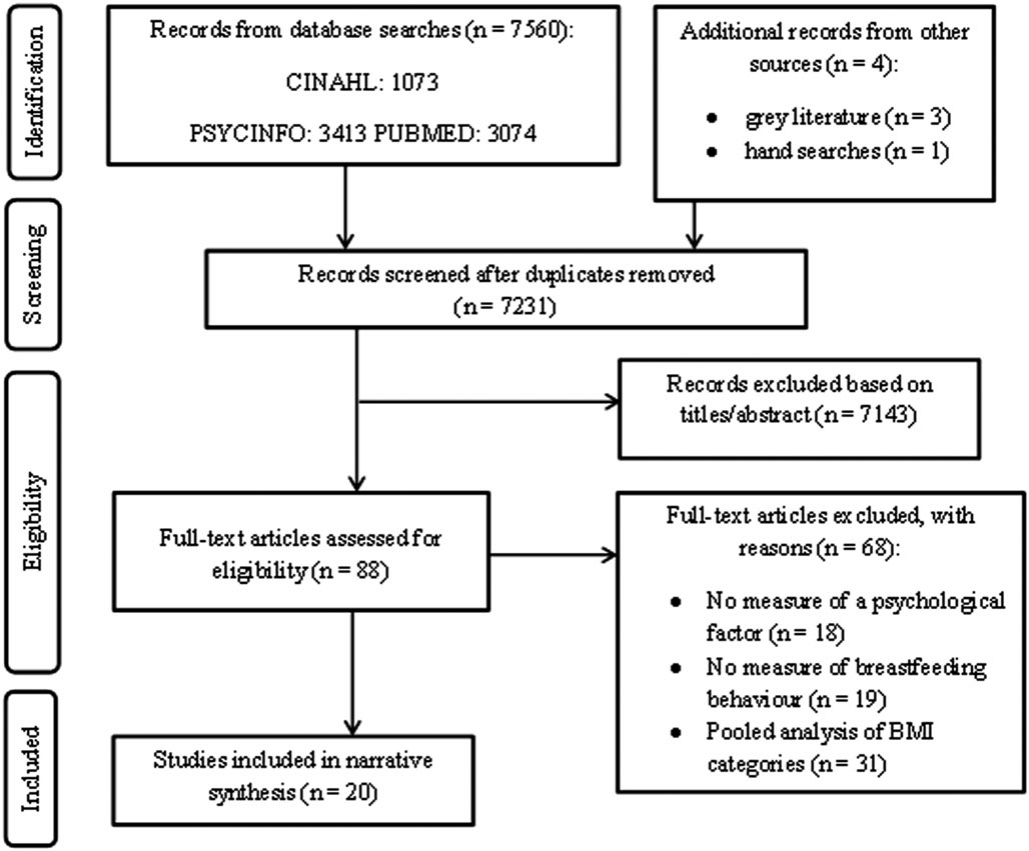
Preferred reporting items for systematic review and meta‐analysis flow diagram of study selection. BMI, body mass index; CINAHL, Cumulative Index to Nursing and Allied Health Literature.

Results from each database were imported into EndNote ×7, and duplicates were removed. Titles and abstracts were screened, excluding those which were not relevant to the research question and/or did not meet the eligibility criteria. At the beginning of this stage, an inter‐rater reliability assessment was conducted, with a second researcher (D. M. S.) assessing and reporting an inclusion/exclusion decision for 10% of the studies identified in the search 28. This was performed by assigning a number to each individual study identified in the search and using a random number generator to select a sample. The decision made by the second researcher was then checked against the first's (S. L.), generating Cohen's kappa statistics. Percentage agreement is not reported because of the large difference in sample sizes and its inability to account for chance agreement 29. There was substantial agreement between researchers, *κ* = 0.74 (95% CI, 0.572 to 0.902), *p* < 0.0005. Consistency in inclusion/exclusion decisions was maintained throughout the remaining studies. Full papers were then retrieved and assessed for inclusion. Again, and in the same way, a second researcher (D. M. S.) assessed and reported an inclusion/exclusion decision for 10% of studies. There was substantial agreement between the researchers, *κ* = 0.78 (95% CI, 0.385 to 1.000), *p* = 0.016. Disagreements were discussed and resolved. The process of study selection is illustrated in a preferred reporting items for systematic review and meta‐analysis flow diagram (Fig. 1).

### Data extraction and quality assessment

Data from the included studies were extracted using a sheet designed for this study (i.e. setting, sample, psychological factor(s), study design, outcome measures and findings). Researchers were contacted for additional information if necessary. All data were anonymized, password protected and only accessible by the research team. The majority of the data used were already in the public domain.

All included studies were assessed for quality using the tool of Hawker *et al*. 30. This tool can assess and therefore allow comparison between a variety of study designs. Using this tool, the ‘abstract and title’, ‘introduction and aims’, ‘method and data’, ‘sampling’, ‘data analysis’, ‘ethics and bias’, ‘results’, ‘transferability or generalizability’ and ‘implications and usefulness’ are awarded a score between 1 and 4 (9 items; total of 36), with higher scoring studies indicating higher quality. For this review, studies scoring ≥28 were considered ‘high’ quality, studies scoring 19–27 were considered ‘fair’ quality and studies scoring 9–18 were considered ‘poor’ quality (see Table 3 for scores). To ensure appraisal quality, two researchers (D. M. S. and S. C.) also completed appraisals for 10% of the included studies, and these were checked against the first's (S. L.). There was moderate agreement between the researchers, *κ* = 0.538 (95% CI, 0.144 to 0.932), *p* = 0.001. Consistency in appraisals was maintained throughout the remaining studies. All but one study 33 fell into the ‘high‐quality’ range.

**Table 3:** Study characteristics of 20 included studies

Reference	Country	Sample	Design	Psychological factor(s)	Breastfeeding behaviour(s)	Quality score*
Bartok *et al*. 31	USA	Women birthing at medical centre	Cohort	Planned duration and general beliefs about BF	Duration	32
Bogen *et al*. 32	USA	Pregnant women volunteers	Observational	Planned IF method	Initiation and duration	34
Chapman *et al*. 23	USA	Pregnant women attending prenatal clinic	RCT	Confidence in ability to BF	Initiation and duration	32
Cordero *et al*. 33	USA	Mothers of macrosomic infants born at a hospital	Cohort	Planned IF method	Initiation	23
Guelinckx *et al*. 34	Belgium	Women birthing at a hospital	Cohort	Planned IF method, belief in breast milk's nutritional adequacy and sufficiency	Initiation and duration	29
Hauff *et al*. 19	USA	Pregnant women volunteers	Cohort	Planned IF method, planned duration, general beliefs about BF, belief about others' IF preferences, confidence in ability to BF and social knowledge	Initiation and duration	32
Hilson *et al*. 18	USA	Pregnant women in hospital database	Observational	Planned duration, general beliefs about BF, body image, confidence in ability to BF, factual knowledge and social knowledge	Duration	30
Jarlenski *et al*. 20	USA	Pregnant women volunteers	Cohort	Planned IF method, general beliefs about BF, belief about others' IF preferences, body image, confidence in ability to BF, BF in social environments, factual knowledge and social knowledge	Initiation and duration	31
Kair *et al*. 35	USA	Women birthing at one of three hospitals	Cohort	General beliefs about BF, belief about others' IF preferences, belief in breast milk's nutritional adequacy and sufficiency	Duration	35
Krause *et al*. 36	USA	Women attending one of three obstetric clinics and volunteers	Observational	Expected outcomes of BF for weight and depressive symptoms	Initiation, duration and intensity	28
Masho *et al*. 37	USA	Women birthing in a hospital	Cohort	Depressive symptoms and stress	Initiation	32
Mehta *et al*. 38	USA	Pregnant women attending a hospital	Cohort	Depressive symptoms, stress and anxiety	Initiation and duration	32
Mok *et al*. 39	France	Women birthing at a hospital	Case–control	Belief in breast milk's nutritional adequacy and sufficiency and BF in social environments	Initiation and duration	31
Newby *et al*. 40	Australia	Pregnant women attending a public event for families	Cohort	Planned IF method, planned duration, confidence in ability to BF and BF in social environments	Duration	31
Ng *et al*. 41	Australia	Women birthing at one of three hospitals	Cohort	Psychological distress	Duration	32
O'Sullivan *et al*. 42	USA	Pregnant women volunteers	Cohort	Planned duration, general beliefs about BF, belief about others' IF preferences, confidence in ability to BF and social knowledge	Duration	32
Swanson *et al*. 43	Scotland	Women birthing at a hospital	Cohort	Body image, psychological distress	BF status	34
Visram *et al*. 44	Canada	Women birthing in one of four hospitals	Cohort	Planned IF method	BF status	32
Zanardo *et al*. 45	Italy	Pregnant women attending a tertiary medical centre	Case–control	Body image	BF at discharge and cessation	28
Zanardo *et al*. 46	Italy	Pregnant women attending a tertiary medical centre	Case–control	Eating disorder symptoms	BF at discharge and cessation	28

* Quality score out of a possible 36.

BF, breastfeed/ing; IF, infant feeding; RCT, randomized controlled trial.

## Results

The search identified 7,564 studies, with 7,231 remaining after duplicates were removed (Fig. 1). Eighty‐eight were reviewed at full text. Twenty studies were included.

### Study characteristics

The characteristics of included studies are summarized in Table 3. Most were conducted in the USA 18, 19, 20, 23, 31, 32, 33, 35, 36, 37, 38, 42, with some in Europe 34, 39, 43, 45, 46, Australia 40, 41 and one in Canada 44. Sample characteristics were reported infrequently; of 20 included studies, 13 reported participant ethnicity or race 19, 20, 23, 31, 32, 33, 35, 36, 37, 38, 39, 40, 42, 9 reported mean age 18, 31, 33, 36, 38, 39, 43, 44, 45 and 5 reported mean BMI 31, 35, 36, 38, 43.

Definitions of breastfeeding behaviours, and the measures used to collect this data, varied between studies. Of 11 studies that reported breastfeeding initiation, nine 19, 20, 23, 32, 34, 36, 37, 38, 39, 43 defined the behaviour as ever receiving breast milk, whereas one 33 recognized initiation as infants receiving ≥50% breast milk feedings upon hospital discharge. Although most studies measured exclusive and any breastfeeding duration 18, 19, 23, 33, 36, 38, 39, 42, 43, two studies 34, 44 measured exclusive breastfeeding only, whereas seven did not 20, 31, 35, 40, 41, 44, 45. Definitions of exclusive breastfeeding varied, depending upon whether the consumption of water, vitamins and medicines were permitted; one study permitted infrequent water consumption 34, four did not 19, 23, 38, 42 and two prohibited all other liquids or solids 19, 43. However, despite definition and measurement variation, of 19 studies comparing women with a BMI ≥30 kg m^−2^ with those with a BMI ≤30 kg m^−2^, women with a BMI ≥30 kg m^−2^ were consistently found to engage less in breastfeeding behaviours 19, 20, 23, 31, 32, 33, 34, 36, 37, 38, 39, 40, 41, 42, 43, 44.

Sixteen psychological factors were identified. The research team discussed these factors in relation to the review aims and grouped together semantically similar factors into five categories: intentions to breastfeed, expectations and beliefs about breastfeeding, psychological well‐being, maternal confidence and breastfeeding knowledge. As the measurement of the psychological factors also varied, a narrative synthesis was produced. Details of measurement are presented in Table 4.

**Table 4:** Descriptions of how psychological factors were measured

Psychological factors	Measures
Intentions to breastfeed	
Planned infant feeding method	Self‐reported infant feeding plan (e.g. breastfeeding, formula and mixed) 19, 20, 32, 33, 34, 40, 44
Planned breastfeeding duration	Self‐reported in months, either as a continuous variable 18, 30, 39 or grouped into ≤6‐, 6‐ to 12‐ or >12‐month categories 19, 42
Expectations and beliefs about breastfeeding	
General beliefs about breastfeeding	Breastfeeding importance rating 20, 31, 42 or scale score of mother's preference towards breastfeeding 18, 19
Belief in breast milk's nutritional adequacy and sufficiency	Reason for noninitiation or cessation 20, 34, 35 or adequate yes/no format 39
Belief about others' infant feeding preferences	Scale scores of others' opinions 19, 42 or as a reason for noninitiation and cessation 20
Expected outcomes of breastfeeding for weight	Scale score of strength of belief 36
Maternal confidence	
Confidence in ability to breastfeed	Scale score of confidence to meet planned duration 19, 40, 42 or BF in different situations 18 or Breastfeeding Self‐Efficacy Scale 23
Breastfeeding in social environments	Scale score of ‘comfortableness in the presence of different groups or in different environments’ 39, 40 or reason for cessation 20
Psychological well‐being	
Body image	Scale score of satisfaction with appearance 18, reason for noninitiation or cessation 20, Multidimensional Body‐Self Relations Questionnaire 43 or Body Uneasiness Test 45
Depressive symptoms	Presence of symptoms in yes/no format 36, 37 or Center for Epidemiologic Studies Depression Scale 38
Stress	Number of stressful life events 37 or Perceived Stress Scale 38
Anxiety	State–Trait Anxiety Inventory 38
Psychological distress	Kessler‐6 Psychological Distress Scale 40 or General Health Questionnaire 43
Eating disorder symptoms	Eating Disorders Inventory‐2 46
Breastfeeding knowledge	
Factual knowledge	True or false questions score 18 or awareness of WHO breastfeeding recommendation 20
Social knowledge	Totalled number of relatives/friends who had breastfed 18, 19, 42

BF, breastfeed/ing; WHO, World Health Organization.

### Intentions to breastfeed

Ten studies 18, 19, 20, 31, 32, 33, 34, 40, 42, 44 reported on infant feeding intentions. This factor was investigated in two forms: planned infant feeding method and planned breastfeeding duration.

#### Planned infant feeding method

Seven studies 19, 20, 32, 33, 34, 40, 44 measured planned infant feeding method. All studies compared women with a BMI ≥30 kg m^−2^ to women with a BMI ≤30 kg m^−2^. Five found that women with a BMI ≥30 kg m^−2^ were significantly less likely to intend to breastfeed 20, 32, 33, 34, 44, suggesting that women with a BMI ≥30 kg m^−2^ are consistently less likely to intend to breastfeed than women with a BMI ≤30 kg m^−2^. As all studies also found that BMI ≥30 kg m^−2^ women were significantly less likely to breastfeed, this suggests that low rates of intention to breastfeed may be associated with their lower rates of breastfeeding.

Three studies 19, 32, 34 investigated whether there was a significant association between intending to breastfeed and breastfeeding behaviour, and all found a direct positive association. Another found extremely high rates of breastfeeding initiation in those who intended (i.e. ranging from 87% to 95% across BMI categories). This again suggests that breastfeeding intention is associated with subsequent breastfeeding behaviour.

#### Planned breastfeeding duration

Five studies 18, 19, 31, 40, 42 measured planned breastfeeding duration. All studies compared the planned breastfeeding duration of women with a BMI ≥30 kg m^−2^ to that of women with a BMI ≤30 kg m^−2^. Only one study reported that women with a BMI ≥30 kg m^−2^ planned to breastfeed for a significantly shorter duration than women with a BMI ≤30 kg m^−2^ 18. This suggests that, of women intending to breastfeed, BMI had no impact on planned breastfeeding duration.

Of four studies reporting no difference in planned breastfeeding duration, all found that women with a BMI ≥30 kg m^−2^ breastfed for a significantly shorter duration than women with a BMI ≤30 kg m^−2^ 19, 31, 40, 42. Despite this, two studies 19, 31 reported a significant positive association between planned and actual duration. However, these results were found by pooling results across BMI categories. A third study 18, when stratifying by BMI, found that although a significant positive association was found for women with a BMI ≤30 kg m^−2^, the association for BMI ≥30 kg m^−2^ women was non‐significant. Therefore, it is unlikely that planned breastfeeding duration is associated with actual breastfeeding duration in women with a BMI ≥30 kg m^−2^.

### Expectations and beliefs about breastfeeding

Nine studies 18, 19, 20, 31, 34, 35, 36, 39, 42 reported on expectations and beliefs about breastfeeding. Several different expectations and beliefs were discussed: general beliefs about breastfeeding, belief about others' infant feeding preferences, belief in breast milk's nutritional adequacy and sufficiency and expected outcomes of breastfeeding for weight.

#### General beliefs about breastfeeding

Five studies 18, 19, 20, 31, 42 examined general beliefs about breastfeeding (i.e. whether breastfeeding was preferable compared with other feeding methods). Across the studies, women with a BMI ≥30 kg m^−2^ preferred breastfeeding. For example, more than 68% of mothers had positive beliefs about breastfeeding 19, and more than 87% rated breastfeeding as at least ‘very important’ 31. However, more than 60% of women who did not initiate rated believing that formula was the same or better than breast milk was an important factor in their decision 20.

No significant differences in beliefs were found between BMI groups. As four studies found that women with a BMI ≥30 kg m^−2^ engaged significantly less in breastfeeding behaviours 19, 20, 31, 42, this suggests that it is unlikely that preferring breastfeeding is associated with behaviour in women with a BMI ≥30 kg m^−2^. One large study found a significant positive association between positive beliefs about breastfeeding and initiation, duration and exclusivity, but this again was found after pooling the results across BMI categories 19. This suggests that it is unlikely that preferring breastfeeding is associated with behaviour in women with a BMI ≥30 kg m^−2^.

#### Belief about others' infant feeding preferences

Four studies 19, 20, 35, 42 investigated beliefs about others' infant feeding preferences. Two studies 20, 35 found that women BMI ≥30 kg m^−2^ were no more likely to report important others wanting to feed their infant as a reason for noninitiation or cessation 20, 35. However, two studies 19, 42 found that women with a BMI ≥30 kg m^−2^ were significantly less likely than women with a BMI ≤30 kg m^−2^ to believe that important others preferred breastfeeding and significantly less likely to breastfeed. This suggests that believing important others prefer breastfeeding as an infant feeding method may be associated with breastfeeding behaviour. This is supported by one study finding a significant, positive association between these two factors 19.

#### Belief in breast milk's nutritional adequacy and sufficiency

Four studies investigated women's belief in the nutritional adequacy and sufficiency of their breast milk 20, 34, 35, 39. All studies found that women with a BMI ≥30 kg m^−2^ were significantly less likely than those with a BMI ≤30 kg m^−2^ to perceive their milk as adequate. As the majority of these studies investigated this factor in terms of contributing to decisions regarding breastfeeding behaviour, this provides strong evidence that lacking belief in breast milk's nutritional adequacy is associated with breastfeeding cessation, despite no study reporting a direct association.

#### Expected outcomes of breastfeeding for weight

One study measured the impact of women with a BMI ≥30 kg m^−2^ expecting breastfeeding to enhance weight loss 36. At 12 months post‐partum, this expectation was significantly negatively correlated with breastfeeding behaviour; higher and increasing expectations from 6 weeks to 12 months were associated with poorer breastfeeding outcomes. This suggests that this expectation may be negatively associated with breastfeeding duration.

### Psychological well‐being

Nine studies 18, 20, 36, 37, 38, 41, 43, 45, 46 explored the impact of psychological well‐being on breastfeeding behaviour. Several symptoms were investigated: body image, depressive symptoms, stress, psychological distress, anxiety and eating disorder symptoms.

#### Body image

Four studies 18, 20, 43, 45 investigated body image, and all found that women with a BMI ≥30 kg m^−2^ had poorer body image than those with a BMI ≤30 kg m^−2^. Two studies found that women with a BMI ≥30 kg m^−2^ were less likely than women with a BMI ≤30 kg m^−2^ to engage in breastfeeding behaviours 20, 43. This suggests that body image may be associated with breastfeeding in women with a BMI ≥30 kg m^−2^.

In support of this, two studies 18, 43 found that body image was positively associated with breastfeeding, with one 18 finding that when entered along with other factors (e.g. shorter planned duration, plans to return to work or school and greater indifference towards breastfeeding), body image attenuated the relationship between obesity and breastfeeding duration. This suggests that it is likely that poorer body image negatively impacts breastfeeding behaviour in women with a BMI ≥30 kg m^−2^.

#### Depressive symptoms

Three studies 36, 37, 38 investigated depressive symptoms in the period surrounding birth. Two studies compared women with a BMI ≥30 kg m^−2^ with those with a BMI ≤30 kg m^−2^ 37, 38; one found that women with a BMI ≥30 kg m^−2^ were significantly more likely to report high levels of depressive symptoms 38. As both studies found these women were significantly less likely to breastfeed, it is unclear whether depressive symptoms are negatively associated with breastfeeding behaviour in women with a BMI ≥30 kg m^−2^.

All three studies conducted association analyses between depressive symptoms and breastfeeding, but results were mixed; one 36 found no relationship between the factors, another found a positive association 37 and one found a negative association, which became non‐significant after accounting for confounding factors 38. This suggests that the relationship between depressive symptoms and breastfeeding behaviour is unclear.

#### Stress

Two studies 37, 38 investigated the impact of stress in the period surrounding the birth. Both studies found that women with a BMI ≥30 kg m^−2^ were more likely to experience stress than those with a BMI ≤30 kg m^−2^, and both also found that this factor was negatively associated with breastfeeding. This suggests that stress levels could explain the lower breastfeeding rates in women with a BMI ≥30 kg m^−2^. However, in one study 38, this relationship became non‐significant after adjusting for confounding factors.

#### Psychological distress

Two studies 40, 43 investigated the impact of psychological distress, defined as a combination of anxiety and depression symptoms surrounding birth. One 40 found that women with a BMI ≥30 kg m^−2^ were significantly more likely to have a medium or high risk of psychological distress at 12 months post‐partum (when many had stopped breastfeeding) but not during pregnancy, whereas the other 43 found no difference between BMI groups. This study 43 found a significant negative association between psychological distress and breastfeeding. Therefore, it is possible that psychological distress is negatively associated with breastfeeding but unlikely that this factor is particularly important to women with a BMI ≥30 kg m^−2^.

#### Anxiety

One study investigated the effect of anxiety in the period surrounding the birth on breastfeeding behaviours 38. This study found that women with a BMI ≥30 kg m^−2^ were significantly more likely to report high levels of anxiety than those with a BMI ≤30 kg m^−2^. It also reported a significant, negative association between anxiety and breastfeeding behaviour. However, this factor became non‐significant after adjusting for confounders, suggesting that it is unlikely that anxiety is associated with breastfeeding behaviour in women with a BMI ≥30 kg m^−2^.

#### Eating disorder symptoms

One study examined eating disorder symptoms 46. This study found that women with a BMI ≥30 kg m^−2^ scored significantly higher than those with a BMI ≤30 kg m^−2^ on body dissatisfaction, ineffectiveness, interoceptive awareness, maturity fears and impulse regulation. However, the study found no differences in breastfeeding rates, suggesting that it is unlikely that eating disorder symptoms are associated with breastfeeding behaviour in women with a BMI ≥30 kg m^−2^.

### Maternal confidence

Eight studies 18, 19, 20, 23, 35, 39, 40, 42 investigated maternal confidence. This was reported in two forms: confidence in ability to breastfeed and breastfeeding in social environments.

#### Confidence in ability to breastfeed

Five studies 18, 19, 23, 40, 42 measured women's confidence in their ability to breastfeed. Two studies reported that women with a BMI ≥30 kg m^−2^ were significantly less likely to have high confidence than those with a BMI ≤30 kg m^−2^ 19, 42, whereas two other studies reported no differences between these groups 18, 40. This may be explained by the extremely high levels of confidence across all participants (e.g. both groups averaging roughly 4.2 out of 5 and >90% of participants reporting high confidence). However, all but one study 18 found that women with a BMI ≥30 kg m^−2^ also engaged less in breastfeeding behaviours. Furthermore, one study found women with a BMI ≥30 kg m^−1^ with higher levels of confidence at 2 weeks were no more likely to be breastfeeding 23. This conflicting evidence makes it difficult to conclude whether having low confidence in ability to breastfeed is associated with decreased breastfeeding in women with a BMI ≥30 kg m^−2^.

One study found a significant positive correlation between confidence and breastfeeding behaviours, but this was found by pooling results across BMI groups 19. Therefore, it is possible that confidence is associated with breastfeeding behaviour in women with a BMI ≥30 kg m^−2^, but firm conclusions cannot be drawn from the current evidence.

#### Breastfeeding in social environments

Three studies 20, 39, 40 investigated women's comfortableness to breastfeed in the presence of others. One study found that women with a BMI ≥30 kg m^−2^ were significantly more likely to feel uncomfortable breastfeeding among close women friends but not in the presence of male friends 40, and another found that women were significantly more likely to feel uncomfortable at 3 months post‐birth but not on the maternity ward or at 1 month 39. One study found no difference between the number of women with a BMI ≥30 kg m^−2^ and those with a BMI ≤30 kg m^−2^ rating not wanting to breastfeed in public as an important reason for cessation 20. As all three of these studies found that BMI ≥30 kg m^−2^ women were less likely to engage in breastfeeding behaviours, it is unlikely that this factor is associated with breastfeeding in women with a BMI ≥30 kg m^−2^.

### Breastfeeding knowledge

Four studies 18, 19, 20, 42 investigated breastfeeding knowledge. This was reported in two forms: factual knowledge and social knowledge.

#### Factual knowledge

Two studies reported on factual breastfeeding knowledge 18, 20. Both studies found no difference in factual knowledge between women with a BMI ≥30 kg m^−2^ and women with a BMI ≤30 kg m^−2^. As only one study found that women with a BMI ≥30 kg m^−2^ were less likely to breastfeed, this suggests that it is unlikely that factual knowledge is associated with breastfeeding in women with a BMI ≥30 kg m^−2^, but neither study confirmed this by conducting an association analysis. However, it is important to note that knowledge levels were not high across all BMI groups; the average score on a breastfeeding knowledge quiz was 6/9 for both groups in one study 18, and only 45% of participants were aware of the 6‐month recommendation in the other 20.

#### Social knowledge

Three studies 18, 19, 42 investigated social knowledge, defined as exposure to breastfeeding through family and friends. Two studies found that women with a BMI ≥30 kg m^−2^ had lower social knowledge (i.e. knew significantly fewer people who had breastfed) than those with a BMI ≤30 kg m^−2^ 19, 42. Both studies also found that women with a BMI ≥30 kg m^−2^ were less likely to breastfeed, suggesting that social knowledge may be associated with breastfeeding behaviour. In support of this, one study 19 found that, even after adjusting for confounders, having a higher level of social knowledge was significantly positively correlated with breastfeeding.

## Discussion

This systematic review adds to current understanding of the influence of psychological factors on breastfeeding behaviours in women with a BMI ≥30 kg m^−2^, which has important implications for reducing obesity rates in both women and children. Almost all included studies found that women with a BMI ≥30 kg m^−2^ were less likely to breastfeed or breastfed for shorter durations than women with a BMI ≤30 kg m^−2^, providing support for previous research 6, 7 and further highlighting the importance of this area.

The review identified several psychological factors that appear to be associated with breastfeeding behaviours in women with a BMI ≥30 kg m^−2^. For example, several studies found that planning to breastfeed was associated with behaviour, but women with a BMI ≥30 kg m^−2^ were less likely than women with a BMI ≤30 kg m^−2^ to do so. This suggests that low rates of breastfeeding intention may explain why fewer women with a BMI ≥30 kg m^−2^ breastfeed. Current care should therefore encourage women with a BMI ≥30 kg m^−2^ to plan to breastfeed. However, as no differences were found between BMI groups for planned breastfeeding duration, and an association between this factor and behaviour was only found for women with a BMI ≤30 kg m^−2^, this suggests that other factors create barriers to breastfeeding maintenance in women with a BMI ≥30 kg m^−2^.

Results suggest having poor body image and lacking belief in breast milk's nutritional adequacy and sufficiency may create barriers and contribute to an explanation of the discrepancy between planned and actual breastfeeding duration in women with a BMI ≥30 kg m^−2^. Included studies consistently found that women with a BMI ≥30 kg m^−2^ had poorer body image and lacked belief in their breast milk's nutritional adequacy and sufficiency, compared with those with a BMI ≤30 kg m^−2^. This may be explained by the elevated focus on their body and, in particular, diet quality during pregnancy to prevent excessive gestational weight gain 47, 48, 49. Although research has shown that milk production can be delayed in women with a BMI ≥30 kg m^−2^ 50, 51 and that milk composition may differ from that of women with a BMI ≤30 kg m^−2^ 52, 53, the WHO still considers breast milk to be the most nutritious milk an infant can receive and recommends that all women breastfeed, regardless of their BMI 5. Therefore, current care could promote positive body image and correct these beliefs, which may reduce barriers and increase breastfeeding rates in women with a BMI ≥30 kg m^−2^.

Two further psychological factors identified may also create barriers to breastfeeding behaviours: belief about others' infant feeding preferences and social knowledge. Included studies found that women with a BMI ≥30 kg m^−2^ were less likely than women with a BMI ≤30 kg m^−2^ to believe that their close friends or family members preferred breastfeeding and were less likely to have friends or family members that had breastfed; both of these factors were associated with breastfeeding behaviours. This finding may reflect the association between having a BMI ≥30 kg m^−2^ and living in areas of economic hardship 54, 55, where breastfeeding rates are already lower 56, 57. Therefore, in line with the theory of planned behaviour 58 and previous research with women living in these areas 59, increasing breastfeeding social norms could increase breastfeeding intention and behaviours in women with a BMI ≥30 kg m^−2^.

Other factors that may create barriers have also been identified in this review, but confirmatory conclusions cannot be drawn. For example, it is possible that expecting that breastfeeding will enhance weight loss has a negative impact on breastfeeding behaviours, but only one study reported on this factor. This was the case for two other factors (i.e. anxiety and eating disorder symptoms), with a further three only reported by two (i.e. stress, psychological distress, factual knowledge). As strong associations between maternal well‐being and factual knowledge and breastfeeding have been found in the general population 60, 61, further research using validated psychological measures and consistent measures of breastfeeding is necessary to determine the true impact of these psychological factors on breastfeeding behaviours in women with a BMI ≥30 kg m^−2^.

Because having confidence in one's ability to breastfeed has been consistently associated with breastfeeding behaviours in women with a BMI ≤30 kg m^−2^ 13, 14, 16, it is surprising that included studies did not provide strong evidence for its role for women with a BMI ≥30 kg m^−2^. However, this may be explained by the majority of these studies measuring confidence in pregnancy, before the women encountered the barriers described earlier. Therefore, it is important for future research to fully investigate the impact of this factor on breastfeeding behaviours in women with a BMI ≥30 kg m^−2^, by examining confidence throughout women's breastfeeding journeys.

Furthermore, it is important to acknowledge that even those psychological factors that were not impacted by BMI may be useful for increasing breastfeeding behaviours in this population. In particular, positive associations were found between general beliefs about breastfeeding and behaviour, despite no difference in beliefs being found across BMI groups. Although the majority of women reported preferring breastfeeding, there was still room for improvement on this factor, and therefore, it may still be useful for increasing breastfeeding in women with a BMI ≥30 kg m^−2^. Similarly, reducing positive beliefs about formula milk (i.e. by reducing advertising) may also increase initiation.

This review had limitations. Firstly, included studies were limited to those written in English, meaning that relevant studies written in other languages may have been excluded. Also, there was a wide variety of measurement of both breastfeeding behaviours and psychological factors. This variation limits comparison between studies and highlights the need for the formation and use of agreed definitions and measures in breastfeeding research. For example, the term ‘breastfeeding maintenance’ should be reserved for those women who breastfeed to any extent for 6 months, in line with the WHO recommendation 5 and the transtheoretical model's definition of maintenance 62. Breastfeeding duration, therefore, would simply denote the length of time a woman breastfed to any extent, with distinctions made between exclusive and any duration. Furthermore, the majority of the studies included were conducted in the USA, with only one conducted in the UK. Although both are classed as developed countries 63, there are important differences in antenatal care in the USA, such as routine weighing at appointments, increased testing for hypertensive disorders, repeated testing for gestational diabetes and weekly foetal testing 48, 49. As care influences women's experiences and beliefs 48, this could limit the applicability of these results to women with a BMI ≥30 kg m^−2^ receiving care in the UK.

This review also has several strengths. Firstly, an extensive scope search was conducted, and pre‐defined inclusion criteria were published, reducing the possibility of researcher bias in study selection 26. Inter‐rater reliability checks were also conducted, further increasing the reliability of the study selection process 64, and a quality appraisal tool was used, which can refine the inclusion criteria and provide possible explanation for conflicting results 28. As all but one of the included studies were high quality, this adds strength to the conclusions drawn.

Several implications and suggestions for future research are generated. Firstly, the results can inform current models of breastfeeding behaviour in women with a BMI ≥30 kg m^−2^. Current healthcare professionals should be aware of the impact of infant feeding intentions, and support should be provided to encourage women with a BMI ≥30 kg m^−2^ to plan to breastfeed and improve their perception of their bodies. Furthermore, once breastfeeding, women should be signposted to breastfeeding support groups to increase their social knowledge and belief that others' prefer breastfeeding and beliefs about the nutritional adequacy of breast milk should be addressed. As many of these psychological factors are under‐researched, future research should focus on conducting longitudinal cohort studies applying validated psychological measures and consistent breastfeeding definitions in order to establish or confirm causality. Furthermore, as breastfeeding rates remain low, interventions utilizing these psychological factors should be developed to increase initiation and duration in women with a BMI ≥30 kg m^−2^. This should be performed in line with the MRC Complex Intervention Framework, combining the relevant theory and evidence base 65. This review suggests that interventions should focus on increasing intentions, promoting positive body image, correcting unrealistic expectations and widening women's social networks. As these results also highlight an intention–behaviour gap, interventions that employ a theoretical framework, which suggests methods of bridging this gap, may be particularly effective.

In conclusion, this review investigated the association of psychological factors with breastfeeding behaviours in women with a BMI ≥30 kg m^−2^. Several psychological factors have been identified, which can be considered and utilized to inform current breastfeeding models, intervention development and antenatal and postnatal care. However, this review highlights that for this population, the role psychological factors play in infant feeding decisions and behaviour is under‐researched, and therefore, more studies are necessary to fully understand their impact. Intervention development is vital to increase breastfeeding and, therefore, prevent and reduce obesity.

## Conflict of interest statement

No conflict of interest was declared.
